# A Novel Biomineralized Collagen Liquid Crystal Hydrogel Possessing Bone-like Nanostructures by Complete In Vitro Fabrication

**DOI:** 10.3390/gels10090550

**Published:** 2024-08-25

**Authors:** Xiaoting Li, Qiaoying Wang, Qingrong Wei

**Affiliations:** 1 School of Medicine and Nursing, Leshan Vocational and Technical College, No. 1336, Middle Section of Qingjiang Avenue, Leshan 614000, China; lixiaoting8180@yeah.net; 2 National Engineering Research Center for Biomaterials (NERCB), College of Biomedical Engineering, Sichuan University, Chengdu 610065, China

**Keywords:** collagen liquid crystal hydrogel, biomineralization, bone-like nanostructures, biomimetic synthesis, in vitro fabrication

## Abstract

The microstructure of bone consists of nano-hydroxyapatite (nano-HA) crystals aligned within the interspaces of collagen fibrils. To emulate this unique microstructure of bone, this work applied two biomimetic techniques to obtain bone-like microstructures in vitro, that is, combining the construction of collagen liquid crystal hydrogel (CLCH) with the application of a polymer-induced liquid precursor (PILP) mineralization process. Upon the elevation of pH, the collagen macromolecules within the collagen liquid crystal (CLC) were activated to self-assemble into CLCH, whose fibrils packed into a long and dense fiber bundle in high orientation, emulating the dense-packed matrix of bone. We demonstrated that the fibrillar mineralization of CLCH, leading to a bone-like nanostructured inorganic material part, can be achieved using the PILP crystallization process to pre-mineralize the dense collagen substrates of CLCH with CaCO_3_, immediately followed by the in situ mineral phase transformation of CaCO_3_ into weak-crystalline nano-HA. The combination of CLCH with the biomineralization process of PILP, together with the mineral phase transformation, achieved the in vitro simulation of the nanostructures of both the organic extracellular matrix (ECM) and inorganic ECM of bone. This design would constitute a novel idea for the design of three-dimension biomimetic bone-like material blocks for clinical needs.

## 1. Introduction

Bone is a functional material comprising organic and inorganic components that are interwoven in a complex arrangement to perform various roles. Its major components include inorganic mineral, consisting of carbonated hydroxyapatite (CHA) nanocrystals, and organic phases, that is, the extracellular matrix of collagen and non-collagenous proteins (NCPs). Mineralized collagen fibril is the basic structural unit of bone tissue [1], which is mineralized with CHA platelets being embedded within the gaps of the fibrils and nearly aligned parallel to the long axis of the collagen fibril [2,3]. The mineralized fibrils further self-assemble into higher structure levels, such as fibers arraying in parallel and rotating across the concentric bone lamellae [1,4]. 

Many studies have developed bone-like collagen–hydroxyapatite composites, mainly utilizing routes such as the simple blending of mineral crystals and collagen; the co-precipitation of mineral nanocrystals with collagen fibrillogenesis; and the immersion of collagen fibrillar scaffolds in simulated body fluid [5], typically using the conventional crystallization reaction. Though these conventional methods have successfully fabricated simple composites with clusters of HA randomly distributed on the surface of collagen fiber, they failed to really achieved intrafibrillar mineralization.

Gower and coworkers [6,7] discovered that acidic polypeptides in mineralization solution can induce an amorphous and liquid-like precursor to the mineral. This polymer-induced liquid precursor (PILP) process could be the base of biomineral morphogenesis in animals [2,8,9,10,11]. Furthermore, it was shown by Li et al. that the intrafibrillar mineralization of collagen can be accomplished via in situ co-deposition synthesis in addition to this PILP process, wherein nanosized HA crystals are also distributed parallel to the long axis within collagen fibrils [2,12,13,14], which almost reproduced the fundamental nanostructures of bone. However, for the biomineralization of a block material of dense collagen hydrogel matrix, the PILP process is more preferable.

Based on this fundamental breakthrough of achieving intrafibrillar mineralization in vitro, some studies applying acidic polymer-mediated mineralization for collagen have been performed on the nanostructure level of individual collagen fibrils [2,15,16], or randomly oriented fibrillar bundles of porous collagen scaffolds [2,3,17,18]. For the mineralization of densely packed collagen substrates, Thula et al. [19] only chose demineralized bone specimens from manatee ribs to serve as the collagen substrates for remineralization using the PILP process. The demineralized bone matrix retained multiple hierarchical levels of collagen organization.

In this study, inspired by PILP investigations and our previous works, we developed a bone-like composite material block, which has biomimetic architectures in both its inorganic and organic nanostructures. This fabrication was designed to be accomplished entirely in vitro. The dense-packed collagen matrix was obtained from the construction of a collagen liquid crystal hydrogel (CLCH) block, which then underwent a PILP process to achieve intrafibrillar mineralization by CaCO_3_ nano-crystals, which were finally transformed into nano-hydroxyapatite in situ. Since the collagen matrix of cortical bone is present in a pattern of cholesteric liquid crystal, from the perspective of complete biomimetic synthesis in vitro, the microstructures of this artificial composite could be close to that of natural bone in the highest degree. Such approach could avoid the cumbersome problem of demineralization–remineralization and the immunogenicity originating from biogenic bone. Simultaneously, this method would also constitute a novel idea for the design of three-dimension biomimetic bone-like material blocks.

## 2. Results and Discussion

### 2.1. Construction of Collagen Liquid Crystal Hydrogel

Collagen (I) is a kind of biomacromolecule which is characterized by spontaneously self-assembling into a fibril network to form hydrogel in vitro under approximate physiological conditions, especially at or above physiological pH value. This self-assembly behavior is the unique native property of collagen macromolecules. Collagen macromolecules can only be dispersed and dissolved in acidic solution.

Collagen macromolecules at low concentration can self-assemble into a hydrogel with a fishnet-like network microstructure. In contrast with the disordered distribution of collagen macromolecules in dilute acidic solution, when the concentration of collagen macromolecules in acidic solution reaches a high enough level, the densely packed collagen macromolecules can align in highly ordered orientation, and this collagen solution at such high concentration is already in solid-like state, which is known as collagen liquid crystalline state.

**Figure 1:**
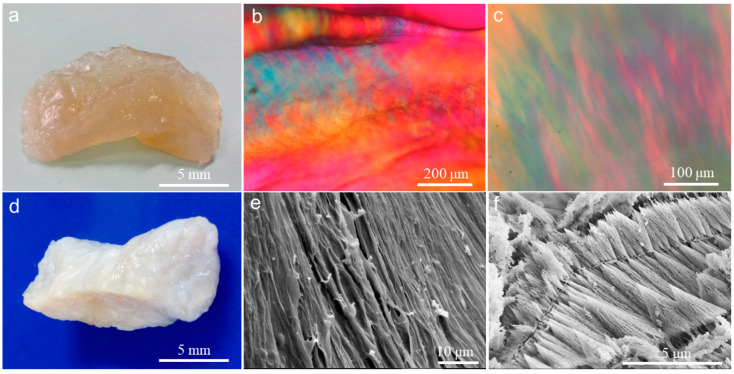
Images showing (**a**) a block of collagen liquid crystal and (**d**) the hydrogel derived from a collagen liquid crystal block. (**b**,**c**) PLM images for collagen liquid crystalline phases, collagen concentration (**b**) at about 200 mg/mL in the central part and (**c**) at about 150 mg/mL in the outer part. SEM images revealing the micromorphology in the (**e**) outer part and (**f**) central part of the CLCH.

Dilute acidic collagen solution is transparent. As the concentration of collagen increases, its transparency decreases. Highly concentrated collagen solution became a solid block of liquid crystal which tended to be translucent (Figure 1a). The PLM images exhibit the optic characteristics of CLC (Figure 1b,c), which were derived from the particular arrangement direction and patterns of the collagen macromolecules in collagen liquid crystalline phases. The optical pattern of CLC is also related to the concentration of the collagen solution. Different degrees of high collagen concentration induce different arrangement directions and patterns of collagen macromolecules. At a concentration of 150 mg/mL, the longitudinal axes of most fibrils had a preferred orientation (Figure 1c). At about 200 mg/mL, all collagen fibrils aligned almost in parallel (Figure 1b).

In ammonia ambience, pH elevation triggered the collagen macromolecules in CLC to self-assemble into fibrillar hydrogel, which was milk-white and opaque (Figure 1d). The microstructures of this CLCH produced from the CLC are shown using SEM (Figure 1e,f). The highly oriented fibrils packed into a long and dense fiber bundle (Figure 1e, Figure S1), whose fibers interconnected with each other, which is very similar to the arrangement and morphology of collagen fiber clusters remaining after the demineralization treatment of dense porcine bone [20,21]. However, the collagen macromolecules in CLC also self-assembled into many short fibers with a coniform or fusiform shape (Figure 1f). These characteristic short fibers were layered according to their orientation, and the orientation on the same layer was highly consistent. The reasons for these two distinct microstructural patterns are likely due to two factors. On the one hand, in this study, the CLC block has a collagen concentration gradient. The concentration in the external liquid crystal region is relatively low, around 150 mg/mL; the concentration in the near-center region is higher, around 200 mg/mL. Consequently, on the other hand, during the transition of CLC to CLCH, there is also a gradient in the penetration of ammonia gas from the outside to the inside of the CLC block. The alkaline gas first reaches the external region where the concentration is relatively low, causing the pH to rise rapidly to above neutral, thus promoting the exhaustive self-assembly of collagen molecules to form a dense hydrogel composed of long and thick fibers, whereas due to the higher collagen concentration in the near-center region of the CLC block, the penetration of alkaline gas is slower, coupled with the gel layer of the external region making the penetration of alkaline gas to the center region even more difficult, resulting in a slow rise in pH in the internal region of the CLC block, and ultimately only reaching a weakly acidic state close to neutral pH. Thus, the self-assembly of these collagen macromolecules was mild, retaining the pattern of the CLC fabric. 

**Figure 2:**
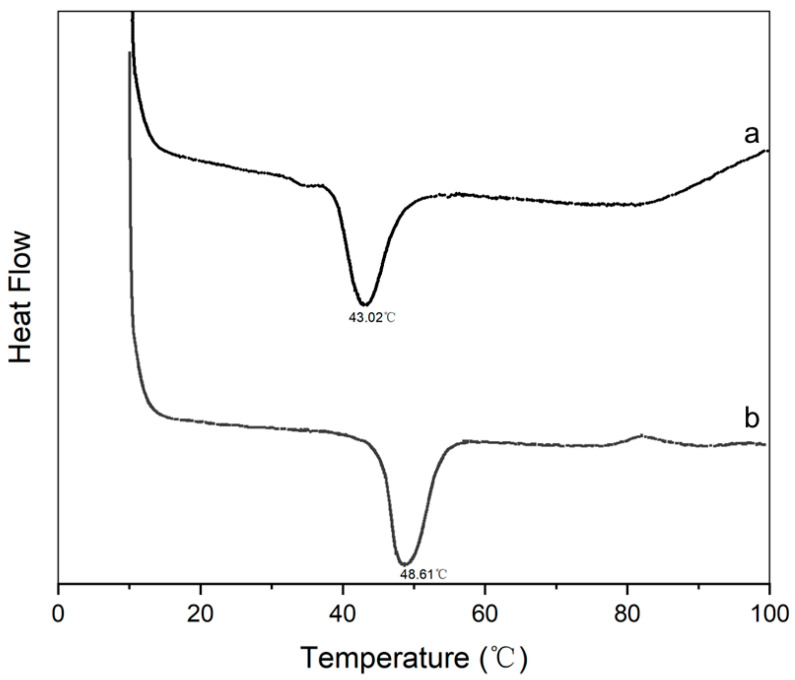
DSC curves of (a) collagen liquid crystal and (b) collagen liquid crystal hydrogel, showing their heat denaturation temperatures.

DSC analyses (Figure 2) revealed that the denaturation temperatures corresponding to the maximal peaks on the DSC curves were around 43.02 °C and 48.61 °C for CLC and CLCH, respectively. The thermal denaturation temperature of CLCH is significantly higher than that of CLC. This is because CLC is essentially a high-concentration solution of collagen molecules, while CLCH is a three-dimensional fibrous network hydrogel solid obtained from the collagen macromolecular self-assembly. Thus, the thermal stability of CLCH is higher than that of CLC.

### 2.2. CaCO_3_ Pre-Mineralization and Mineralization of CLCH by Nano-HA via CaCO_3_ Conversion

Collagen is the most important biomacromolecule in regulating bone development. However, it is obviously not the only one responsible for controlling bone mineralization since the body mostly consists of collagenous tissues which never mineralize [22,23]. Similarly, over the past few decades, the mineralization of type-I collagen in vitro by many researchers did not completely reproduce the collagen–mineral microstructure of bone at the nanoscale level [24,25]. Therefore, the role of the non-collagenous proteins (NCPs) associated with bone or dentin is deemed vital for the crystal nucleation and growth of biomineralization [26,27]. Most of these proteins are highly acidic. These acidic polymers were demonstrated to produce a precursor process for biomineralization, which is named PILP process, an inherently different crystallizing mechanism from conventional solution crystallization [6,28]. This PILP process can produce crystal morphologies close to that of native bone.

**Figure 3:**
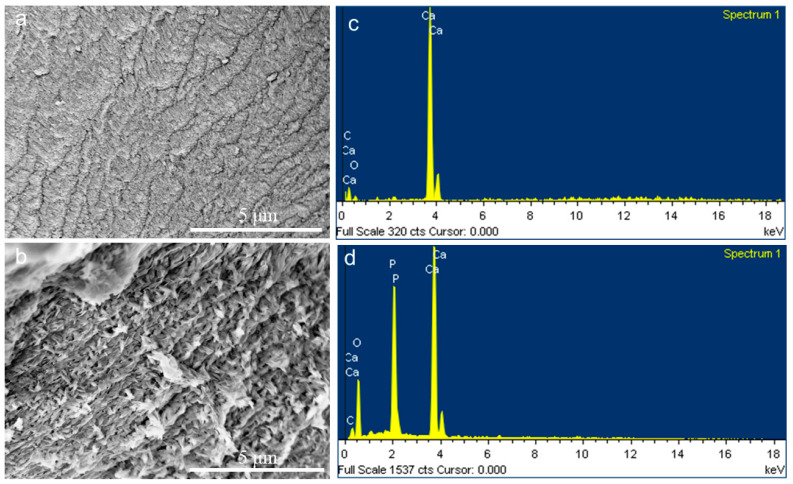
SEM images of (**a**) CaCO_3_-pre-mineralized CLCH using the PILP process and (**b**) HA-mineralized CLCH derived from CaCO_3_-pre-mineralized CLCH in 0.1 M phosphate solution for 3 days. EDS of (**c**) CaCO_3_-pre-mineralized CLCH using the PILP process and (**d**) HA-mineralized CLCH derived from CaCO_3_-pre-mineralized CLCH in 0.1 M phosphate solution for 3 days.

**Figure 4:**
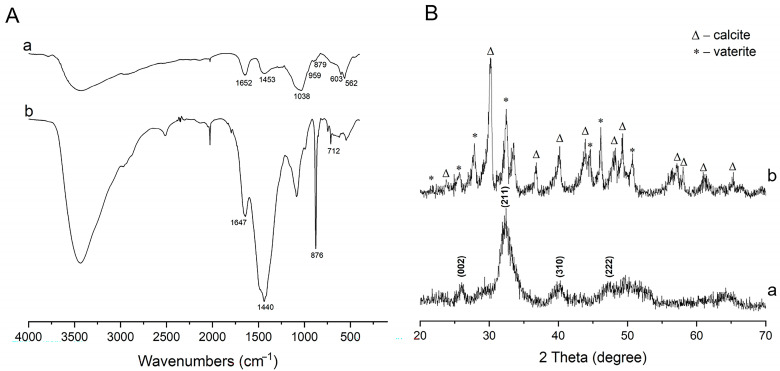
(**A**) FTIR and (**B**) XRD spectra analyzed from (**a**) CaCO_3_-mineralized CLCH using the PILP process and (**b**) HA-mineralized CLCH derived from CaCO_3_-pre-mineralized CLCH in 0.1 M phosphate solution for 3 days.

Although the inorganic phase in the natural biomineralization of vertebrates is calcium phosphate rather than calcium carbonate, this study achieved the pre-mineralization of CLCH with calcium carbonate via the biomimetic mineralization mechanism of PILP. A continuous and dense inorganic layer had completely encased the dense collagen fibers of the CLCH (Figure 3a), whose high calcium content was confirmed by means of EDS (Figure 3c). This inorganic layer enriched in calcium was identified as typical calcium carbonate mineral phase using FTIR (Figure 4A) and XRD (Figure 4B), comprising CaCO_3_ vaterite and calcite. The absorption bands at 1440 (v_3_), 876 (v_2_), and 712 cm^−1^ (v_4_) were derived from the characteristic vibrations of carbonate in the pre-mineralized CLCH. Both the orientation and the contour of the mineralized collagen fibers are easily discriminable (Figure 3a).

When the PILP process is applied to mineralize a collagen matrix, an extremely distinct form is produced, known as the amorphous precursor phase. This phase is inherently liquid and not a crystalline structure, thereby exhibiting characteristics of space filling. These traits enable it to seep into the various structural levels of collagen fiber bundles. Through the iterative application of the PILP method, the amorphous liquid precursor can fully permeate the collagen matrix. Finally, these amorphous precursors coalesce and crystallize to form a highly mineralized mineral coating.

Based on our previous related research [29,30], as a template, this existing calcium carbonate mineral phase was transformed into a calcium phosphate mineral phase through soaking in phosphate solution at physiological temperature for a period of time. FTIR and XRD spectra demonstrated that this phase transformation caused the CaCO_3_ mineral to be thoroughly substituted by the needle-like or sheet-like nanocrystals of HA (Figure 4A,B), which continuously encased the dense collagen fibers of CLCH in an orientation along the long axis (Figure 3b). Figure S2 shows the cross-section of the mineralized CLCH block, which was similar to the cross-section morphology of demineralized dense porcine bone [20,21]. The EDS analyses revealed that the nano-HA minerals attained a Ca/P ratio value of 1.495 (Figure 3d). Such carbonated apatite is close to biologically generated HA, which facilitates reabsorption for remodeling within tissues [31,32]. The amide I band was present in both pre-mineralized CLCH with CaCO_3_ (1647 cm^−1^) and final-mineralized CLCH with HA (1652 cm^−1^). The bands at 1038 (v_3_), 959 (v_1_), 603 (v_4_), and 562 (v_4_) cm^−1^ represent the PO_4_ ^3−^ groups of HA [33]. Figure 4B(a) also reveals the characteristic peaks of 002, 211, 310, and 222 attributing to weak-crystalline HA. These findings indicate that the transformed inorganic phase within the mineralized CLCH was primarily composed of a HA crystalline phase, which completely encapsulated the collagen fibers. Thereby, the mineralized CLCH is composed of interpenetrating networks of organic–inorganic phases, achieving the biomimetic mineralization for CLCH with nano-HA.

**Figure 5:**
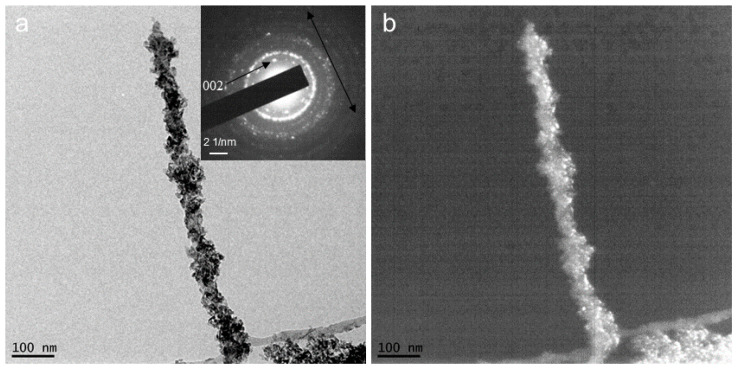
Electron micrographs of mineralized collagen fibril sampled from the mineralized CLCH in Figure 3b. (**a**) TEM bright field image of a mineralized collagen fibril; the inset shows the SAED pattern of this mineralized collagen fibril. (**b**) Selected area diffraction pattern of the middle of the mineralized fibril in (**a**), indicating that the mineral is hydroxyapatite. Note that the 002 plane is oriented along the long axis of the collagen fibril (long arrow).

TEM analyses revealed the mineralization details of the collagen fibrils within the biomineralized CLCH. The inorganic nanocrystals had grown in clusters densely wrapping the collagen fibrils (Figure 5a and S3). From the SAED pattern (Figure 5b), the mineral content had been identified as HA with weak crystallinity, which was consistent with the analyses of XRD.

## 3. Conclusions

From a biomimetic perspective, a bone-like composite material simulating both the organic and inorganic microstructures of cortical bone was successfully synthesized in vitro. The construction of a collagen liquid crystal hydrogel block was designed to provide a dense-packed collagen matrix similar to the organic matrix of bone tissue. By means of intrafibrillar pre-mineralization with CaCO_3_ nano-crystals via the PILP process, followed by mineral phase transformation in situ, this CLCH matrix received biomineralization with nano-hydroxyapatite. 

This study highlights the proof-of-concept design of imitating bone material by means of a preparation method entirely in vitro, via the combination of CLCH with the PILP process and mineral phase transformation. In this way, the desired composite was obtained. But further investigations will be necessary to explore the effects of the biomimetic microstructures of the biomineralized CLCH on the biological behaviors of osteogenic-related cells.

## 4. Materials and Methods

Type-I collagen was provided by our laboratory using pepsin digestion of calf skin. (NH_4_)_2_CO_3_, Na_2_HPO_4_·12H_2_O, anhydrous CaCl_2_, and other chemicals (Kermel Ltd., Chengdu, China) were of analytical grade purity and were used directly without further treatment. Polyacrylic acid (PAA, Mw = 5500) was from Sigma-Aldrich, (St. Louis, MO, USA). Water (18 MΩ/cm) from a Milli-Q Synthesis System (Merck Millipore, Burlington, NJ, USA) was used in all of our experiments. All collagen mentioned in this study refers to type-I collagen.

### 4.1. Preparation of Three-Dimensional Collagen Liquid Crystal and Its Hydrogel

Acidic collagen solution at a concentration of 5 mg/mL in 0.5 M acetic acid was initially sheared and concentrated using a membrane ultrafiltration system to form a relatively thick collagen solution with a concentration of 10–15 mg/mL, which was then further concentrated by means of reverse dialysis against polyethylene glycol (20 kDa) solution in 0.5 M acetic acid [34]. This procedure achieved a very high concentration of collagen solution of about 150–200 mg/mL, which was already in a collagen liquid crystal (CLC) state. To maintain the highly ordered pattern of a collagen liquid crystalline microstructure, the fibrillogenesis of collagen macromolecules was initiated by increasing the pH value of the collagen liquid crystal block in ammonia vapor and maintaining it for 18 h, so as to avoid introducing aqueous solvent into the liquid crystal [35]. Thus, a compact hydrogel possessing a liquid crystalline arrangement was obtained, which was named collagen liquid crystal hydrogel (CLCH). 

### 4.2. Pre-Mineralization for CLCH by CaCO_3_ via PILP Process

After being thoroughly washed in ultrapure water, the CLCH slices (0.5 cm × 0.35 cm × 0.35 cm) were mineralized with calcium carbonate via a PILP process. The samples of two CLCH slices were first soaked into 5.0 mL of filtered 0.2 M calcium chloride solution overnight, then soaked into 5.0 mL of PAA solution (300 µg/mL) overnight, and finally placed into a polystyrene Petri dish with a diameter of 3.5 cm, to which 0.2 mL calcium chloride solution, 1.0 mL PAA solution, and 1.0 ultrapure water were added to obtain a final volume of 4 mL. The dish was sealed up with stretched Parafilm, into which several holes were punched to allow for the vapor diffusion of the carbonate species into the solution. The crystallizing dish was put in a closed desiccator, along with three small beakers (5 mL) filled with powder of (NH_4_)_2_CO_3_. The beakers were also sealed up with stretched Parafilm and had one or two holes punched in the center for allowing for the diffusion of the decomposition products into the crystallizing solutions containing calcium–PAA. The desiccator was then placed at 4 °C for three days, at which time the CLCH samples were taken from the solution and rinsed with ultrapure water, and then again subjected to the same mineralization phase of three days in fresh calcifying solutions, and the reaction was allowed to continued. Consequently, the CLCH samples were mineralized up to three cycles with a total crystallizing time of 9 days. One of these samples was stored at −80 °C for freeze-drying and characterization; another sample was subjected to mineral transformation from CaCO_3_ to HA. 

### 4.3. Intrafibrillar Mineralization of CLCH by Nano-HA Derived from CaCO_3_ Templet Conversion

The mineral transformation from CaCO_3_ to HA for the pre-mineralized CLCH (PM-CLCH) was achieved by soaking PM-CLCH in Na_2_HPO_4_ solutions (0.1 M, 37 °C) replaced every 24 h for 3 days. The ratio of gels to phosphate solution was about 1:20 (*w*/*v*). The mineralized CLCH samples obtained in this way were thoroughly rinsed in ultrapure water to wash away redundant phosphates and residues and then lyophilized for characterization. Herein, as a pre-mineral, CaCO_3_ acts as a mineral template.

### 4.4. Polarizing Light Microscopy 

To characterize the textures of the collagen liquid crystal obtained in this way, the sample was cut into thin slices (1 cm × 1 cm × 0.1 cm) firstly, then placed between a slide and a coverslip, and analyzed under a polarized light microscope (PLM, DMLP, Leica, Wetzlar, Germany) at room temperature.

### 4.5. Differential Scanning Calorimetry

The specimens of 5.0–10 mg CLC or CLCH were sealed in aluminum pans for differential scanning calorimetry (DSC, TA Q2000, Newcastle, DE, USA) analyses. Temperature was raised from 10 °C to 100 °C at 1 °C/min under a high pure nitrogen atmosphere. Then, the samples were cooled down to 10 ℃ at 3 °C/min after balancing for 5 min. The temperature at a peak maximum was named denaturation temperature.

### 4.6. Scanning Electron Microscopy

The microstructures of non-mineralized and mineralized CLCH were revealed under a field emission scanning electron microscope (FESEM, S-4800 Hitachi, Tokyo, Japan) equipped with an energy-dispersive spectrum (EDS). The specimens prepared by lyophilization were attached to conductive adhesive directly with a Au sputtering coat for SEM and without Au coating for EDS. The conditions for performing the FESEM were a voltage of 5 kV, a vacuum degree of 10^−6^ Pa, and maintaining this vacuum condition throughout the whole observation period.

### 4.7. Transmission Electron Microscopy

The mineralization state of the collagen fibrils by mineral nanocrystals was explored using transmission electron microscopy (TEM, FEI, Tecnai G2 F20-TWIN, Hillsboro, OR, USA). The sample was crushed into fine powder in liquid nitrogen. Then, a small drop of ethanol was added to the powder to form a slurry, which was then transferred to carbon-coated copper grids for air drying. Selected area electron diffraction (SAED) was also conducted on the mineralized fibril for identifying the inorganic phase. The conditions for performing the TEM were a voltage of 200 kV, a vacuum degree of 10^−6^ Pa, and always maintaining this vacuum condition throughout the whole observation period. 

### 4.8. X-Ray Diffraction and Fourier-Transform Infrared Spectroscopy

After lyophilization (samples were pre-frozen overnight at −80 °C, then lyophilized in a freeze dryer with a cold trap temperature of −99 °C and a vacuum of 16.0 Pa), the mineralized CLCH samples were smashed and ground into powders whose composition phases were analyzed via X-ray diffraction (XRD, X’Pert Pro, PHILIPS). Fourier-transform infrared spectroscopy (FTIR) spectra for the mineralized CLCH samples were detected using an analyzer (NEXUS 670, Thermo Electron, Waltham, MA, USA) in the wavenumber range of 4000–400 cm^−1^ with 4 cm^−1^ as the resolution. 

## Data Availability

The original contributions presented in the study are included in the article, and further inquiries can be directed to the corresponding authors.

## Supplementary Materials

The following supporting information can be downloaded at: https://www.mdpi.com/article/10.3390/gels10090550/s1, Figure S1: SEM image revealing the micromorphology in the outer part of the CLCH block; Figure S2: SEM image from the cross-section of the HA-mineralized CLCH block derived from CaCO_3_-pre-mineralized CLCH soaked in 0.1 M phosphate solution for 3 days; Figure S3: TEM bright field images of the mineralized collagen fibril sampled from the biomineralized CLCH.
