# Supplementary material for: A Novel Biomineralized Collagen Liquid Crystal Hydrogel Possessing Bone-like Nanostructures by Complete In Vitro Fabrication
Source: Gels. 2024 Aug 25;10(9):550. doi: 10.3390/gels10090550 (PMC11431544; doi:10.3390/gels10090550)
Supplement: Supplementary file 1 [file gels-10-00550-s001.zip › gels-3162779-supplementary.pdf]

Supplementary Information

A novel biomineralized collagen liquid-crystal hydrogel possessing bone-like nanostructures by complete *in vitro* fabrication

Xiaoting Li[1], Qiaoying Wang[1,*] and Qingrong Wei[2,*]

[1] School of Medicine and Nursing, Leshan Vocational and Technical College, No. 1336, Middle Section of Qingyijiang Avenue, Shizhong District, Leshan city, 614000, P. R. China

[2] National Engineering Research Center for Biomaterials (NERCB); College of Biomedical Engineering, Sichuan University, Chengdu 610065, P. R. China

[*]Authors to whom any correspondence should be addressed.

E-mail: wangqi1854@gmail.com; qingrongwei@scu.edu.cn

Results

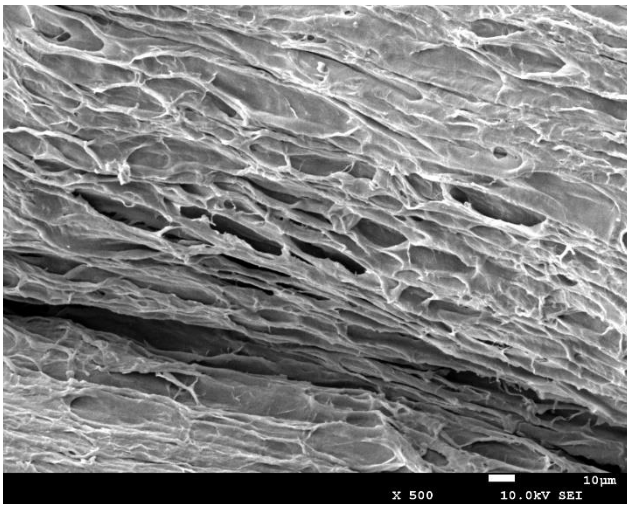

Figure S1. SEM image revealing the micromorphology in the outer part of the CLCH block.

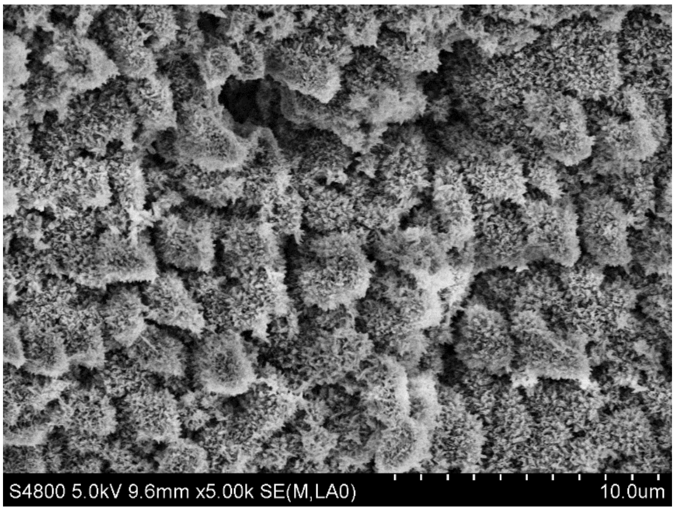

Figure S2. SEM image from the cross section of HA mineralized CLCH block derived from $CaCO_3$ pre-mineralized CLCH soaked in 0.1M phosphate solution for 3 days.

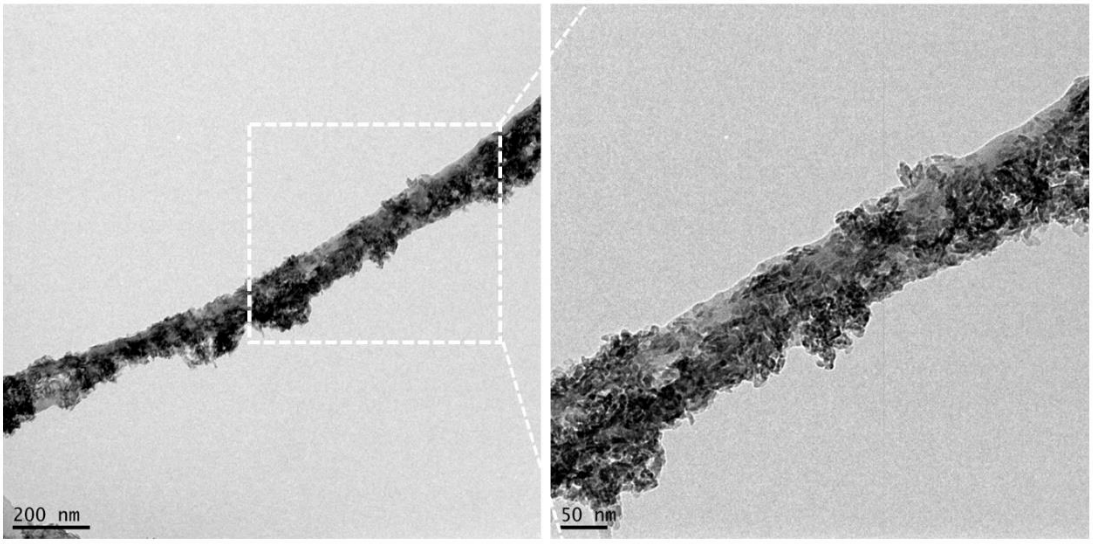

Figure S3. TEM Bright Field images of the mineralized collagen fibril sampled from the biomineralized CLCH.
